# Evaluation of knowledge‐based planning models for male pelvic CBCT‐based online adaptive radiotherapy on conventional linear accelerators

**DOI:** 10.1002/acm2.14464

**Published:** 2024-07-19

**Authors:** Riley C. Tegtmeier, Edward L. Clouser, Brady S. Laughlin, Diego A. Santos Toesca, Mattison J. Flakus, Sara Bashir, Christopher J. Kutyreff, Dean Hobbis, Daniel P. Harrington, Jennifer L. Smetanick, Nathan Y. Yu, Carlos E. Vargas, Sarah E. James, Jean‐Claude M. Rwigema, Yi Rong

**Affiliations:** ^1^ Department of Radiation Oncology Mayo Clinic Arizona Phoenix Arizona USA; ^2^ Department of Radiation Oncology Washington University School of Medicine in St. Louis St. Louis Missouri USA

**Keywords:** adaptive radiotherapy, CBCT, knowledge‐based planning, prostate radiotherapy, RapidPlan

## Abstract

**Purpose:**

To assess the practicality of employing a commercial knowledge‐based planning tool (RapidPlan) to generate adapted intact prostate and prostate bed volumetric modulated arc therapy (VMAT) plans on iterative cone‐beam computed tomography (iCBCT) datasets.

**Methods and materials:**

Intact prostate and prostate bed RapidPlan models were trained utilizing planning data from 50 and 44 clinical cases, respectively. To ensure that refined models were capable of producing adequate clinical plans with a single optimization, models were tested with 50 clinical planning CT datasets by comparing dose‐volume histogram (DVH) and plan quality metric (PQM) values between clinical and RapidPlan‐generated plans. The RapidPlan tool was then used to retrospectively generate adapted VMAT plans on daily iCBCT images for 20 intact prostate and 15 prostate bed cases. As before, DVH and PQM metrics were utilized to dosimetrically compare scheduled (iCBCT Verify) and adapted (iCBCT RapidPlan) plans. Timing data was collected to further evaluate the feasibility of integrating this approach within an online adaptive radiotherapy workflow.

**Results:**

Model testing results confirmed the models were capable of producing VMAT plans within a single optimization that were overall improved upon or dosimetrically comparable to original clinical plans. Direct application of RapidPlan on iCBCT datasets produced satisfactory intact prostate and prostate bed plans with generally improved target volume coverage/conformality and rectal sparing relative to iCBCT Verify plans as indicated by DVH values, though bladder metrics were marginally increased on average. Average PQM values for iCBCT RapidPlans were significantly improved compared to iCBCT Verify plans. The average time required [in mm:ss] to generate adapted plans was 06:09 ± 02:06 (intact) and 07:12 ± 01:04 (bed).

**Conclusion:**

This study demonstrated the feasibility of leveraging RapidPlan to expeditiously generate adapted VMAT intact prostate and prostate bed plans on iCBCT datasets. In general, adapted plans were dosimetrically improved relative to scheduled plans, emphasizing the practicality of the proposed approach.

## 1 INTRODUCTION

The concept of adaptive radiotherapy (ART) was introduced in the late 1990s to enable modification of the original treatment plan by capitalizing on supplementary anatomical and functional information acquired throughout the treatment course.^1^ Plan adaptation may involve revision of plan contours to accommodate anatomical variations or dynamic adjustment of clinical objectives and constraints to be more or less aggressive as appropriate.^2^, ^3^ Notably, failure to account for concurrent spatial variation and gross changes to tumor anatomy (e.g., shrinkage) as visualized on daily images during fractionated treatments can influence the fidelity of plan delivery.

For the prostate region (both intact and post‐operative), studies have demonstrated that physiological changes in bladder and rectum volume may contribute to significant clinical target volume (CTV) displacements as large as ∼2 cm.^4^, ^5^, ^6^, ^7^ While advancements in cone‐beam computed tomography (CBCT)‐based image guidance techniques have largely addressed translational variations,^8^ independent motion of the prostate and seminal vesicles (intact prostate) and the highly deformable and mobile nature of the CTV (prostate bed) may lead to compromised alignment during treatment.^9^, ^10^, ^11^ To mitigate the potential of inadequate target volume coverage and loss of tumor control due to spatial/anatomical variation, generous planning target volume (PTV) margins of 5–7 mm have been utilized in conventional radiotherapy to ensure sufficient coverage.^11^ However, utilization of larger margins may contribute to higher rates of reported patient toxicities due to elevated organ‐at‐risk (OAR) doses.^11^ Therefore, such margins are unsuitable for modern hypo‐fractionated treatment regimens. To combat this, for example, improved image‐guidance for target localization and pre‐treatment alignment through the emergence of magnetic resonance imaging (MRI)‐guided radiotherapy has allowed for margin reductions (to 2–3 mm) for both intact^12^ and post‐operative^11^ prostate stereotactic body radiotherapy cases, demonstrating the benefit of reduced acute toxicities for OARs. However, the reduction of treatment margins requires accurate monitoring of dose delivery and/or plan adaptation when necessary. Consequently, online ART (oART), in which the treatment plan is adjusted based on images acquired immediately prior to a treatment fraction while the patient remains in the treatment position, has emerged as a promising solution by demonstrating significant clinical benefits for both intact prostate^10^, ^13^, ^14^, ^15^, ^16^, ^17^, ^18^, ^19^, ^20^, ^21^ and prostate bed patients.^11^, ^22^

Although the concept of ART has been present for several decades and has been shown to offer considerable advantages regarding treatment accuracy and outcome, its effective clinical implementation continues to be hampered by technical and logistical limitations, as this process introduces time‐ and resource‐intensive complexities into the clinical workflow.^2^, ^23^ As a result, the integration of oART capabilities is presently confined to institutions equipped with specialized MRI‐guided (e.g., Elekta Unity, Elekta AB, Stockholm, Sweden) or CT‐based (e.g., Halcyon/Ethos, Varian Medical Systems, Palo Alto, CA and Radixact/ClearRT, Accuray, Inc., Sunnyvale, CA) platforms and software. This institution has therefore commenced a preliminary investigation into the feasibility of utilizing the Eclipse Scripting Applications Programming Interface (ESAPI—Varian Medical Systems) to develop a semi‐automated, CBCT‐based oART workflow for conventional C‐arm linear accelerators (linacs). By leveraging tools more commonly available across the broader radiation oncology community, the ultimate aim is to offer the potential for more accessible and widespread use of oART in the clinic.

A crucial component of the proposed workflow involves the rapid generation and optimization of refined treatment plans based on the daily anatomy provided by the pre‐treatment CBCT setup image. For this purpose, knowledge‐based planning (KBP), a technique that leverages historical planning data to predict achievable plan quality given the current patient's anatomical information, offers a potentially viable solution. By automating the generation of ideal optimization constraints tailored to specific patient anatomies, KBP assists in improving plan quality by reducing inter‐clinician variation and provides an efficient means by which to produce clinically acceptable treatment plans with minimal workflow requirements and reduced planning duration.^24^, ^25^ Nearly a decade ago, a commercial KBP tool, RapidPlan, was integrated into the Eclipse treatment planning system (Varian Medical Systems). This tool utilizes a statistical model generated from a library of clinically approved plans, employing principal component analysis to discern robust correlations between geometric parameters (e.g., percentage overlap between an OAR and target volume) and dosimetric metrics (e.g., the fraction of an OAR subject to a specific dose).^26^ Provided this information, RapidPlan utilizes dose‐volume histogram (DVH) predictions to formulate individualized optimization criteria. The performance of this tool has been assessed against manually optimized clinical plans for various treatment sites and techniques, including intact prostate.^24^, ^25^, ^26^, ^27^, ^28^, ^29^ These studies have indicated that the use of RapidPlan induces improved or comparable plan quality while significantly increasing consistency and efficiency in the planning process, highlighting the potential utility of this tool for the rapid generation of clinically acceptable plans in lieu of dedicated oART planning software.

The objective of this investigation, therefore, was to assess the viability of employing institution‐specific intact prostate and prostate bed RapidPlan models to produce, via a single optimization, clinically acceptable plans on CBCT datasets as required by the oART workflow under development. To ensure satisfactory clinical performance, the trained models first underwent initial validation via retrospective comparison with manually optimized plans on planning CT (pCT) datasets as commonly documented in the literature.^24^, ^25^, ^26^, ^28^, ^29^ Following validation of the models, an adaptive scenario was retrospectively simulated for a select cohort of intact prostate and prostate bed patients previously treated at this institution. This process involved a comparison of appropriate plan quality metrics between the original plan on the pCT dataset, the original plan as propagated to the daily CBCT image, and an adapted plan optimized directly on the CBCT image through the use of the appropriate RapidPlan model. To the knowledge of the authors, there have been no prior comprehensive studies evaluating the fidelity of plan generation on CBCT datasets via direct application of the RapidPlan tool.

## 2 METHODS AND MATERIALS

### 2.1 Model configuration, validation, and initial testing

The study described herein received approval by this institution's ethical research review board (IRB ID: 22‐002186). Institution‐specific RapidPlan models were formulated for both intact prostate and post‐prostatectomy patients utilizing a database comprised of volumetric‐modulated arc therapy (VMAT) treatment plans (on pCT) previously administered within the clinic. Initial training for each model included data extracted from 50 patients and 44 patients, respectively. Models were configured adhering to manufacturer guidelines as detailed in the literature.^25^, ^30^ Note that all pCT images utilized within the study were acquired with standard *Pelvis* scanning protocols (120 kVp, variable mAs based on patient size, 2 mm slice thickness) on a commercial CT simulation unit (Siemens SOMATOM Definition AS RT, Siemens AG, Munich, Germany). Exclusion criteria for plan selection included patients treated to multiple dose levels and/or with nodal involvement (single or chain), individuals with hip prostheses, and/or cases presenting with abnormal anatomical variations (e.g., hernias, lymphoceles, etc.). Among the 50 patients included for training the intact prostate model, 20 cases featured a rectal balloon, 16 utilized spacing hydrogels, and 48 included implanted fiducials in the target. Variable parameters were utilized within these training plans, with energies of 10X (*n* = 22) and 10XFFF (*n* = 15), and prescriptions of 3500cGy in 5 fractions (*n* = 20), 6000cGy in 20 fractions (*n* = 14), and 7000cGy in 28 fractions (*n* = 12) being most common. For patients included in training the prostate bed model, a rectal balloon was present in 10 cases, while energies of 6X (*n* = 12) and 10X (*n* = 24), and prescriptions of 3000−3200cGy in 5 fractions (*n* = 9) and 6400−6600cGy in 32−33 fractions (*n* = 28) were most prevalent. All plans utilized in any manner throughout this study consisted of two full treatment arcs with variable collimator rotation. Volumes‐of‐interest considered for model definition and training were the CTV, PTV, bladder, rectum, femoral heads, penile bulb, and bowel (large and small). Based on institutional guidelines, standard CTV‐to‐PTV expansions were 3 mm (with only 2 mm in the posterior direction for additional rectum sparing) for intact prostate and 5 mm (uniform) for prostate bed. All intact prostate targets included the proximal seminal vesicles. For optimal model performance, optimization structures including control rings of various expansions from the target volume, target/OAR overlap volumes (for bladder and rectum), and OAR avoidance structures (rectum) are recommended as these were included when training the models. These optimization volumes are either manually segmented by the dosimetrist (control rings, rectal avoidance structures) or generated automatically when running in‐house software developed for the intended CBCT‐based oART workflow (target/OAR overlap volumes). However, the presence of all aforementioned optimization structures is not strictly required for satisfactory plan generation as they are typically assigned relatively low priorities by the model regardless—nonetheless, they offer the ability to further refine the plans if deemed necessary.

Model validation was performed in part by inspection of plans flagged as statistically significant outliers for the user by the software and by evaluation of the quality of treatment plans selected for the training. Iterative adjustments were implemented as necessary to optimize model parameters until performance was deemed satisfactory by physician review. To obtain an unbiased estimate of this final model performance, an additional 50 patient plans (on pCT) were used to test each model. The testing dataset for the intact prostate model once again included cases with rectal balloon (*n* = 44), hydrogel spacers (*n* = 11), and implanted fiducials (*n* = 43), whereas energies of 6X (*n* = 14), 10X (*n* = 13), and 10XFFF (*n* = 18), and prescriptions of 3500cGy in 5 fractions (*n* = 22) and 6000cGy in 20 fractions (*n* = 17) were most common. Of the 50 patients utilized to test the prostate bed model, 14 received treatment with a rectal balloon, with energies of 6X (*n* = 29) and 10X (*n* = 16), and prescriptions of 6400−6800cGy in 32−34 fractions (*n* = 30) being most prevalent. Plans within the training/testing datasets were selected solely based on the availability of clinically relevant data devoid of any disqualifying criteria as listed above, and their inclusion in either dataset was determined by the recency of the treatment. The overarching objective throughout the configuration process was to develop models capable of generating optimal treatment plans for a variety of dose prescriptions, fractionation schemes, and treatment energies. Of note, for intact prostate and prostate bed plan generation at this institution, standard constraint templates for plan evaluation are applied regardless of the fractionation scheme or prescribed dose. Additionally, empirical data from clinical practice has indicated that the utilization or omission of a rectal balloon and/or hydrogel spacer has little to no influence on model performance. Consequently, separate models were not trained for patients with or without such rectal‐sparing devices.

### 2.2 Plan evaluation metrics

Within the testing datasets, plans generated via RapidPlan were compared to manually optimized plans as treated clinically through a variety of evaluation criteria. This comparison aimed to ensure that with a single optimization, models performed in a manner dosimetrically equivalent (or similar) to that observed for clinical plans generated and iteratively optimized by the dosimetrist. As part of the testing and validation process, comparison metrics were selected based on DVH criteria outlined in the RTOG0126 (intact prostate) and RTOG0534 (prostate bed) protocols with the addition of complementary PTV and low‐dose DVH points. Since the relevant RTOG protocols were based on conventional fractionation (and considering patients in the testing datasets were treated with a variety of fractionation regimens), the equivalent dose in 2 Gy fractions (EQD2) was utilized for all dose‐based metrics to provide a means of more objective comparison. For all EQD2 calculations, alpha/beta ratios of 10 and 3 Gy were assumed for all target and normal tissues (bladder, rectum, femoral heads), respectively. Evaluated PTV metrics included D_98%_ (near minimum dose), D_95%_, D_mean_, and D_2%_ (near maximum dose). Additionally, the homogeneity index (HI–defined as [D_2%_—D_98%_]/D_prescription_) and conformity index (CI–defined as [V_100%_/V_PTV_]) were calculated. Metrics for OARs included V_70Gy,EQD2_, V_65Gy,EQD2_, V_60Gy,EQD2_, V_50Gy,EQD2_, V_40Gy,EQD2_, V_30Gy,EQD2_, D_mean_, and D_0.03cc_ (bladder and rectum), and D_mean_ and D_1.0cc_ (femoral heads).

In addition to the aforementioned DVH parameters, a single plan quality metric (PQM) value, referring to a user‐defined metric intended to provide a means of plan comparison and based on a modified formalism pioneered by Nelms et al.,^31^ was also adopted as a global measure of plan quality. This comparison tool was built through a list of additional sub‐metrics (largely independent of those listed above) that schematically represented the goals of the treatment. Sub‐metrics utilized for PQM analysis (Table 1) were based on those provided in clinical constraint templates employed as the primary means of quantitative plan evaluation at this institution and were assessed via EQD2 conversion as before to accommodate variable fractionation schemes (note that an alpha/beta value of 6 Gy was assumed for the large and small bowel). The scoring mechanism within this tool was dependent on the volume‐type associated with each sub‐metric. A majority of PTV objectives (2 of 3) were pass/fail, and by meeting the specified constraint the corresponding sub‐metric received maximum points (100 points were available for each PTV criterion). This approach attempted to reduce masking plan flaws through normalization. For goals associated with OARs (and the remaining PTV sub‐metric), scoring was implemented on a gradient scale with a maximum of either 50 or 80 points (100 for the PTV sub‐metric). If some minimum dose threshold was achieved, full points were allotted, whereas if some maximum dose value was exceeded, no points were awarded (see Table 1). For intermediate values between maximum and minimum dose constraints, scoring was based on a weighted percentage of the attained value relative to that value corresponding to the maximum score. The final PQM value was the summation of the scores obtained for each individual sub‐metric (note that this scoring was automated through internal scripting). For intact prostate plans generated on pCT images, the maximum achievable score was 1030. For prostate bed plans, 950 total points were available (the V_70Gy,EQD2_ metric for the bladder was not evaluated). Overall, this tool offers a prompt and objective method by which to compare the quality of different plans within a single patient or between patients based on clinically relevant sub‐metrics.

**TABLE 1:** Summary of the scoring mechanism within the plan quality metric (PQM) tool. Included are each sub‐metric and the range of possible scores. Dose‐based metrics (e.g., D_95%_) are relative to the prescription dose, while volume‐based metrics (e.g., V_70Gy,EQD2_) are relative to the total volume of the corresponding organ. Parameters listed are for the intact prostate PQM algorithm, with any variation within the prostate bed algorithm noted in italics.

	Planning goal sub‐metric	Maximum points available	Value to receive maximum points	Value to receive no points
PTV	D_95%_ ≥ 100%	100	100%	<100% or >101%
	D_0.03cc _< 105−110%	100	<105%	>110%
	Min ≥ 90%	100	≥90%	<90%
Bladder	V_70Gy, EQD2_ < 10% *(NA)*	80	0%	>10% (*NA*)
	V_65Gy, EQD2_ < 50% *(60%)*	50	0%	>50% (*60%*)
	D_0.03cc_ < 103−105% *(108%)*	80	≤103%	>105% (*108%*)
Rectum	V_70Gy, EQD2_ < 15−25%	80	0%	>25%
	V_65Gy, EQD2_ < 50−60%	80	0%	>60%
	D_0.03cc_ < 103−105% *(108%)*	50	≤103%	>105% (*108%*)
LFH	V_50Gy, EQD2_ < 1cc	50	0 cc	>1 cc
RFH	V_50Gy, EQD2_ < 1cc	50	0 cc	>1 cc
L Bowel	D_0.03cc_ < 60 Gy	80	0 Gy	>60 Gy
S Bowel	V_45Gy, EQD2_ < 100 cc	50	0 cc	>100 cc
	D_0.03cc_ < 55 Gy	80	0 Gy	>55 Gy
	**Total points available**	**1030 (*950*)**		

Abbreviations: L, large; LFH, left femoral head; PTV, planning target volume; RFH, right femoral head; S, small.

As an aside to mitigate potential confusion, note that within this manuscript, the terms “DVH metrics/parameters” and “PQM sub‐metrics/values” are intended to refer to the two distinct quantitative evaluations and are not used interchangeably (though the sub‐metrics included within the PQM analysis are technically DVH parameters as well). For further clarification, the sub‐metrics compiled to generate the PQM scores were selected at the discretion of the physicians within the prostate group at this institution and are constraints for achieving the most superior plan quality based on physician experience and clinical practice patterns. Therefore, the PQM sub‐metrics as provided in Table 1 may or may not correspond to the DVH metrics listed for the referenced RTOG protocols (the PQM sub‐metrics are generally tighter constraints, whereas the DVH metrics per RTOG protocols are typically the minimum acceptable values). Within the proposed oART process, the singular PQM scores as detailed will serve as the principal mode of quantitative plan assessment/comparison. However, to provide a more exhaustive comparative analysis for the purposes of this study, the evaluation of the additional RTOG‐based DVH parameters as described was included as well.

For plan comparisons within the model testing datasets, statistical analysis of RTOG‐based DVH metrics was performed through a two‐sided *t*‐test with a significance level of 0.05 (*p*‐values < 0.05 indicate a significant difference in the mean values), while PQM values were compared via the Wilcoxon signed‐rank test (with a significance level of 0.05).

Furthermore, additional plan parameters, including the total number of monitor units (MU) necessary for plan delivery and the final plan normalization value needed to achieve the desired target coverage (D_95% _≥ 100%), were analyzed to help further assess similarities/dissimilarities between the various plans.

### 2.3 CBCT‐based plan generation and comparison

Following configuration, validation, and testing phases, the models were then used to generate plans directly on CBCT datasets in an effort to simulate tasks within an oART workflow. For this process, final fraction on‐treatment imaging data from an additional 20 intact prostate cancer patients without nodal or metastatic involvement and 15 post‐prostatectomy patients previously treated at this institution was utilized. Images were acquired employing iterative CBCT (iCBCT), a novel image reconstruction infrastructure available on conventional TrueBeam linacs (Varian Medical Systems, Inc., Palo Alto, CA). Details pertaining to this advanced imaging solution are provided in the literature.^32^, ^33^ All iCBCT images utilized within the study were acquired with the standard *Pelvis* scanning protocol (125 kVp, 1080 mAs, 2.5 mm slice thickness). Within the 20 intact prostate cases, a rectal balloon, hydrogel spacer, and implanted fiducials were present in 8, 6, and 17 cases, respectively. The breakdown of applied energies and prescriptions was 6X (*n* = 4), 6XFFF (*n* = 3), 10X (*n* = 8), and 10XFFF (*n* = 5), and 3500cGy in 5 fractions (*n* = 8), 6000cGy in 20 fractions (*n* = 6), and 7000cGy in 28 fractions (*n* = 6). For the 15 prostate bed cases, a balloon was present in 3 instances, with energies of 6X (*n* = 5) and 10X (*n* = 10), and prescriptions of 6400cGy in 32 fractions (*n* = 6) and 6600cGy in 33 fractions (*n* = 9) utilized for the clinical plans. The relevant target volumes (CTVs) were propagated to the iCBCT images following rigid registration to the pCT image based on clinical image guidance procedures, with modifications to these volumes occurring as needed by a trained physician. Upon physician review and modification of target contours as would be performed in the proposed workflow, these volumes were then peer‐reviewed by a trained physicist. Lastly, appropriate CTV‐to‐PTV margins were applied following confirmation of these structures. The OARs (bladder, rectum, and femoral heads) were contoured via an institution‐specific deep learning‐based auto‐segmentation (DLAS) model trained specifically on iCBCT datasets and validated in the literature,^34^ ensuring that all contours adhered to institutional segmentation guidelines as would be utilized to delineate these structures on pCT. Prior to plan generation, the accuracy of these contours was again verified by a physicist at this institution, and modifications were made as necessary. Note that average revision times for OARs returned by this auto‐segmentation model are detailed in a previous study.^34^

For each patient identified, two plans were generated for comparative analysis. First, the original clinical plan as optimized on the pCT image was projected onto the corresponding iCBCT image to evaluate the fidelity of dose delivery as initially intended (iCBCT Verify plan). The resulting dose distribution was then compared to that obtained with a single optimization via direct application of the appropriate RapidPlan model on the iCBCT dataset, with all plan parameters (energy, prescription, field setup, etc.) identical to those of the original clinical plan and all plans normalized to a target coverage of D_95% _= 100%. The couch was added as appropriate on each iCBCT image to ensure the most accurate DVH generation and comparison. Relevant RTOG‐based DVH metrics and the PQM tool were utilized to compare plan quality as before. Importantly, the large and small bowel were not delineated on iCBCT images. Given that no cases with nodal involvement were included within this evaluation (and it was verified prior to plan generation that for the iCBCT‐based cases the bowel structures were well outside the treated area), achieving the constraints associated with these volumes (Table 1) was of minimal concern and had little impact on the plan optimization/generation process. Consequently, it was deemed to be of little benefit to put forth additional effort to train the OAR DLAS model to include these structures or to delineate them manually in this particular study. As such, maximum attainable PQM scores were reduced to 820 for intact prostate and 740 for prostate bed.

Finally, to further evaluate the feasibility of utilizing this tool within an oART workflow in which temporal efficiency is imperative, the time needed to generate the iCBCT Verify and iCBCT RapidPlan plans within the in‐house software was recorded. It should be noted that all timing metrics presented in this study pertain exclusively to the plan generation/optimization process. The additional temporal requirements for DLAS/propagation of OARs and target volumes, as well as for the subsequent review and revision of such structures prior to plan generation, have been detailed in a prior study.^34^

For comparison of data from three groups (clinical plan, iCBCT Verify plan, iCBCT RapidPlan), a repeated Analysis of Variance (ANOVA) single factor test, coupled with a Tukey honestly significant difference post‐hoc test, was utilized to perform statistical analysis of the relevant RTOG‐based DVH metrics (both tests were performed with a significance level of 0.05). PQM values were compared via the Friedman test paired with the Nemenyi post‐hoc test (again with significance levels of 0.05). All statistical analysis in the study was performed with the appropriate macro functions available via the Excel spreadsheet software (Microsoft Corp., Redmond, Washington).

## 3 RESULTS

### 3.1 Plan evaluation metrics for model testing datasets

#### 3.1.1 RTOG‐based DVH metrics

Results for the RTOG‐based DVH metrics utilized for comparison between the pCT‐based clinical and RapidPlan plans are presented in Table 2. For nearly all PTV metrics (excluding HI) within the intact prostate cohort, statistically significant differences were observed between the results (*p*‐values < 0.01). However, these disparities do not appear to hold clinical significance provided the small magnitude of variation. While most bladder and rectum metrics demonstrated no statistically significant variation, application of the intact prostate RapidPlan model marginally increased the bladder hot spot (D_0.03cc_—*p*‐value < 0.01) while improving V_40Gy, EQD2_, V_30Gy, EQD2_, and D_mean_ metrics for the rectum (*p*‐values < 0.01) relative to clinical plans. All femoral head metrics exhibited statistically significant differences (*p*‐values < 0.01), but as before, the clinical significance of this variation is expected to be negligible. Similar trends were observed within the prostate bed testing cohort, though fewer statistically significant variations within PTV metrics and no significant differences within bladder and rectum metrics were demonstrated.

**TABLE 2:** Mean RTOG‐based dose‐volume histogram (DVH) metric values for all cases within the model testing cohorts. Values are reported to one standard deviation. Dose‐based metrics (e.g., D_98%_) are relative to the prescription dose, while volume‐based metrics (e.g., V_70Gy,EQD2_) are relative to the total volume of the corresponding organ. *P*‐values shown in bold indicate those that demonstrate no statistically significant variation.

	DVH parameter	Intact prostate (*n* = 50)			Prostate bed (*n* = 50)		
		Clinical plan	RapidPlan	*p*‐value*	Clinical plan	RapidPlan	*p*‐value*
PTV	D_98%_ [%]	99.5 ± 0.5	99.3 ± 0.3	<0.01	99.5 ± 0.6	99.1 ± 0.3	<0.01
	D_95%_ [%]	100.3 ± 0.3	100.0 ± 0.0	<0.01	100.3 ± 0.4	100.0 ± 0.1	<0.01
	D_mean_ [%]	102.0 ± 0.3	101.4 ± 0.2	<0.01	102.0 ± 0.4	102.0 ± 0.3	**0.56**
	D_2%_ [%]	103.8 ± 0.6	103.4 ± 0.4	<0.01	104.1 ± 0.6	104.1 ± 0.5	**0.90**
	HI	0.04 ± 0.02	0.04 ± 0.01	**0.47**	0.05 ± 0.02	0.05 ± 0.01	**0.06**
	CI	0.96 ± 0.02	0.95 ± 0.01	<0.01	0.96 ± 0.02	0.95 ± 0.01	<0.01
Bladder	V_70Gy, EQD2_ [%]	2.4 ± 2.8	2.9 ± 3.0	**0.36**	–	–	–
	V_65Gy, EQD2_ [%]	3.7 ± 3.6	4.4 ± 3.9	**0.38**	11.5 ± 11.1	10.8 ± 10.6	**0.73**
	V_60Gy, EQD2_ [%]	5.0 ± 4.4	5.7 ± 4.7	**0.44**	14.9 ± 11.8	13.9 ± 11.4	**0.67**
	V_50Gy, EQD2_ [%]	7.6 ± 6.2	8.5 ± 6.9	**0.53**	19.8 ± 12.2	18.3 ± 11.9	**0.55**
	V_40Gy, EQD2_ [%]	11.5 ± 8.6	12.5 ± 9.8	**0.57**	23.2 ± 13.9	21.1 ± 13.3	**0.43**
	V_30Gy, EQD2_ [%]	17.3 ± 11.5	18.6 ± 12.8	**0.58**	27.7 ± 16.1	24.6 ± 14.9	**0.32**
	D_mean_ [%]	20.1 ± 9.5	21.6 ± 10.4	**0.46**	31.7 ± 15.2	29.4 ± 14.6	**0.44**
	D_0.03cc_ [%]	103.6 ± 1.1	104.5 ± 1.1	<0.01	104.8 ± 1.0	105.0 ± 0.8	**0.24**
Rectum	V_70Gy, EQD2_ [%]	1.5 ± 1.8	1.1 ± 1.3	**0.23**	–	–	–
	V_65Gy, EQD2_ [%]	2.8 ± 2.6	2.2 ± 1.9	**0.17**	6.2 ± 4.6	5.9 ± 4.4	**0.79**
	V_60Gy, EQD2_ [%]	4.1 ± 3.2	3.2 ± 2.4	**0.14**	9.4 ± 5.7	9.0 ± 5.3	**0.70**
	V_50Gy, EQD2_ [%]	6.9 ± 4.6	5.4 ± 3.3	**0.07**	14.9 ± 6.4	14.6 ± 5.4	**0.75**
	V_40Gy, EQD2_ [%]	11.5 ± 6.5	8.6 ± 4.3	<0.01	19.9 ± 7.8	19.4 ± 6.3	**0.69**
	V_30Gy, EQD2_ [%]	19.8 ± 8.9	14.0 ± 4.7	<0.01	26.6 ± 10.1	25.1 ± 7.7	**0.40**
	D_mean_ [%]	25.7 ± 6.1	21.8 ± 3.9	<0.01	33.9 ± 8.0	31.8 ± 6.9	**0.17**
	D_0.03cc_ [%]	100.0 ± 6.2	99.9 ± 6.1	**0.90**	104.3 ± 0.7	104.4 ± 0.6	**0.57**
LFH	D_mean_ [%]	16.8 ± 3.5	19.4 ± 3.4	<0.01	20.2 ± 4.0	23.0 ± 4.5	<0.01
	D_1cc_ [%]	37.6 ± 6.1	40.7 ± 4.9	<0.01	42.4 ± 5.9	48.9 ± 6.8	<0.01
RFH	D_mean_ [%]	17.8 ± 3.5	20.2 ± 3.4	<0.01	20.2 ± 3.8	23.2 ± 4.5	<0.01
	D_1cc_ [%]	38.1 ± 6.3	42.0 ± 5.3	<0.01	42.8 ± 5.5	48.4 ± 6.1	<0.01

*Notes*: *P‐values based on two‐sided *t‐*test with an alpha value of 0.05, null hypothesis that there is no statistically significant difference between the mean values—if *p*‐value is <0.05, reject the null hypothesis.

Abbreviations: CI, conformity index; HI, heterogeneity index; LFH, left femoral head; PTV, planning target volume; RFH, right femoral head.

#### 3.1.2 PQM values

**FIGURE 1:**
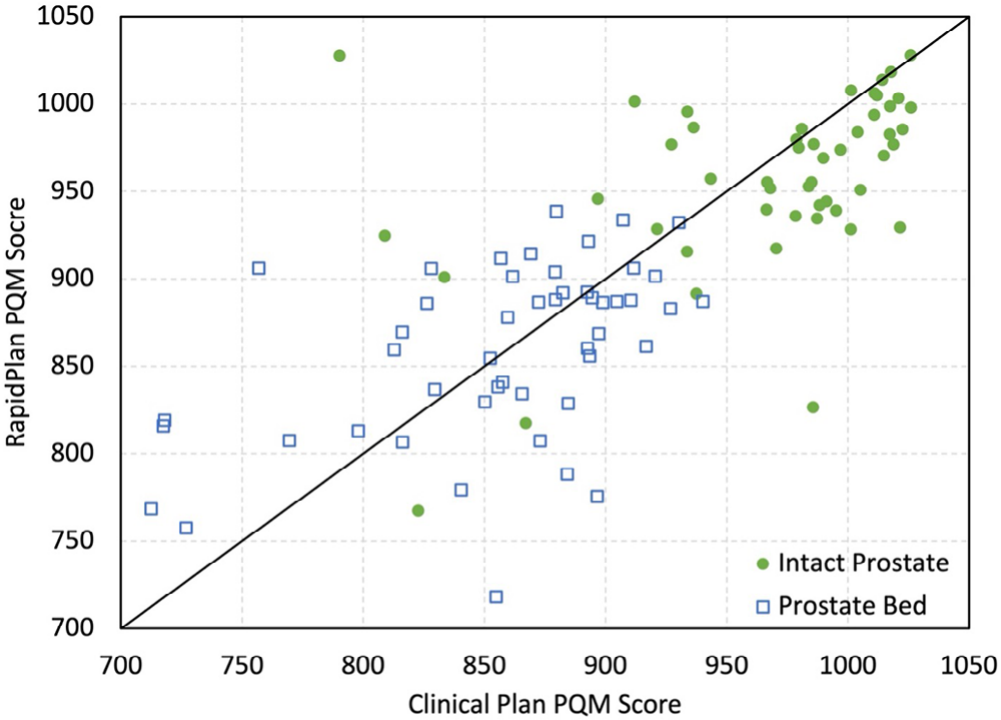
Plan quality metric (PQM) scoring comparison between clinical and RapidPlan plans in the model testing datasets. Data points below the line indicate a higher PQM score for the clinical plan, while data points above the line indicate a higher PQM score for the plan generated via RapidPlan. Maximum achievable scores were 1030 for intact prostate and 950 for prostate bed.

Mean PQM values obtained for all testing patients within each cohort are detailed in Tables 3 and 4, respectively, while a visual representation of these results is presented in Figure 1. For the intact prostate cohort, clinical plans scored higher in 70% of cases (35/50), with a mean total score of 968.1 ± 58.6 compared to 957.6 ± 51.5 for plans generated via RapidPlan. However, the Wilcoxon signed‐rank test indicated a non‐significant variation (*p*‐value of 0.39). In total, 7 failures (no points awarded) were observed for clinical plans (6 corresponding to target coverage sub‐metrics), while 6 failures were present within RapidPlan results (all associated with bladder dosage). For the prostate bed cohort, plans generated via RapidPlan scored higher in 54% of cases (27/50) with an average score of 859.7 ± 50.1 compared to 853.5 ± 63.0 for clinical plans, though variation in these mean values was not statistically significant (*p*‐value of 0.61). As before, 7 failures in total were present for clinical plans (6 related to PTV coverage), while RapidPlan results demonstrated 5 failures (3 for PTV, 2 for large bowel sub‐metrics).

**TABLE 3:** Mean plan quality metric (PQM) values for intact prostate cases (*n* = 50) within the model testing cohort. Values for the sub‐metrics and the corresponding sub‐metric scores are reported to one standard deviation. Failures indicate instances in which no points were awarded.

	Sub‐metric	Clinical plan (intact prostate)			RapidPlan (intact prostate)		
		Mean value	Mean score	Failures	Mean value	Mean score	Failures
PTV	D_95%_ [%]	100.3 ± 0.3	94.0 ± 24.0	3	100.0 ± 0.0	100.0 ± 0.0	0
	D_0.03cc_ [%]	104.9 ± 0.6	96.4 ± 7.2	0	105.2 ± 0.6	94.0 ± 9.1	0
	Min [%]	93.3 ± 2.7	94.0 ± 24.9	3	93.8 ± 1.4	100.0 ± 0.0	0
Bladder	V_70Gy, EQD2_ [%]	0.7 ± 2.1	74.6 ± 16.7	1	0.8 ± 2.4	74.4 ± 17.2	2
	V_65Gy, EQD2_ [%]	3.7 ± 3.6	46.3 ± 3.6	0	4.5 ± 3.9	45.5 ± 3.9	0
	D_0.03cc_ [%]	103.6 ± 1.1	59.5 ± 20.6	0	104.5 ± 1.2	42.9 ± 25.8	4
Rectum	V_70Gy, EQD2_ [%]	1.5 ± 1.8	80.0 ± 0.0	0	1.5 ± 1.9	80.0 ± 0.0	0
	V_65Gy, EQD2_ [%]	2.8 ± 2.6	76.2 ± 3.5	0	3.4 ± 2.7	75.5 ± 3.6	0
	D_0.03cc_ [%]	100.0 ± 6.2	48.7 ± 5.1	0	100.0 ± 4.6	48.7 ± 4.1	0
LFH	V_50Gy, EQD2_ [cc]	0.0 ± 0.0	50.0 ± 0.0	0	0.0 ± 0.0	50.0 ± 0.0	0
RFH	V_50Gy, EQD2_ [cc]	0.0 ± 0.0	50.0 ± 0.0	0	0.0 ± 0.0	50.0 ± 0.0	0
L Bowel	D_0.03cc_ [Gy_EQD2_]	6.2 ± 7.9	70.1 ± 11.5	0	7.1 ± 8.2	68.6 ± 12.1	0
S Bowel	V_45Gy, EQD2_ [cc]	0.0 ± 0.0	50.0 ± 0.0	0	0.0 ± 0.0	50.0 ± 0.0	0
	D_0.03cc_ [Gy_EQD2_]	1.0 ± 1.4	78.2 ± 2.3	0	1.2 ± 1.8	77.9 ± 3.0	0
	Total score (*p*‐value* = 0.39)		**968.1 ± 58.6**			**957.6 ± 51.5**	

*Notes*: *P‐value based on Wilcoxon signed‐rank test with an alpha value of 0.05, null hypothesis that there is no statistically significant difference between the mean values—if *p*‐value is <0.05, reject the null hypothesis.

Abbreviations: L, large; LFH, left femoral head; PTV, planning target volume; RFH, right femoral head; S, small.

**TABLE 4:** Mean plan quality metric (PQM) values for prostate bed cases (*n* = 50) within the model testing cohort. Values for the sub‐metrics and the corresponding sub‐metric scores are reported to one standard deviation. Failures indicate instances in which no points were awarded.

	Sub‐metric	Clinical plan (prostate bed)			RapidPlan (prostate bed)		
		Mean value	Mean score	Failures	Mean value	Mean score	Failures
PTV	D_95%_ [%]	100.3 ± 0.4	94.0 ± 24.0	3	100.0 ± 0.1	100.0 ± 0.0	0
	D_0.03cc_ [%]	105.5 ± 1.0	86.8 ± 16.5	0	105.5 ± 0.6	89.7 ± 10.8	0
	Min [%]	92.2 ± 2.8	94.0 ± 24.0	3	92.5 ± 1.7	94.0 ± 24.0	3
Bladder	V_65Gy, EQD2_ [%]	11.5 ± 11.1	40.4 ± 9.3	0	10.8 ± 10.6	41.0 ± 8.9	0
	D_0.03cc_ [%]	104.8 ± 1.1	56.1 ± 13.2	0	105.0 ± 0.8	53.2 ± 10.6	0
Rectum	V_70Gy, EQD2_ [%]	0.6 ± 1.0	80.0 ± 0.0	0	0.7 ± 1.1	80.0 ± 0.0	0
	V_65Gy, EQD2_ [%]	6.2 ± 4.6	71.8 ± 6.2	0	5.9 ± 4.4	72.1 ± 5.9	0
	D_0.03cc_ [%]	104.3 ± 0.7	38.9 ± 5.1	0	104.4 ± 0.6	38.3 ± 4.8	0
LFH	V_50Gy, EQD2_ [cc]	0.0 ± 0.0	50.0 ± 0.0	0	0.0 ± 0.0	50.0 ± 0.0	0
RFH	V_50Gy, EQD2_ [cc]	0.0 ± 0.0	50.0 ± 0.0	0	0.0 ± 0.0	50.0 ± 0.0	0
L Bowel	D_0.03cc_ [Gy_EQD2_]	11.7 ± 16.0	64.4 ± 20.1	1	12.1 ± 17.6	64.1 ± 12.0	2
S Bowel	V_45Gy, EQD2_ [cc]	0.0 ± 0.0	50.0 ± 0.0	0	0.0 ± 0.0	50.0 ± 0.0	0
	D_0.03cc_ [Gy_EQD2_]	1.9 ± 3.3	77.2 ± 4.8	0	1.8 ± 3.2	77.4 ± 4.6	0
	Total score (*p*‐value* = 0.61)		**853.5 ± 63.0**			**859.7 ± 50.1**	

*Notes*: *P‐value based on Wilcoxon signed‐rank test with an alpha value of 0.05, null hypothesis that there is no statistically significant difference between the mean values—if *p*‐value is <0.05, reject the null hypothesis.

Abbreviations: L, large; LFH, left femoral head; PTV, planning target volume; RFH, right femoral head; S, small.

#### 3.1.3 Additional plan quality metrics

On average, intact prostate plans generated via RapidPlan demonstrated slight increases in total MUs relative to clinical plans (higher by 1.9% ± 15.8%; min = −33.5%, max = 33.8%, med = 2.9%), though 44% of plans (22/50) exhibited a decrease in MUs. RapidPlan results for prostate bed revealed a slight decrease in total MUs on average (lower by −6.1% ± 11.9%; min = −27.3%, max = 17.5%, med = −6.2%), while 32% of plans (16/50) demonstrated an increase in total MUs.

Average plan normalization values were 99.9 ± 0.3 (min = 98.9, max = 100.7, med = 100.0) for intact prostate clinical plans, 99.4 ± 0.2 (min = 98.9, max = 99.8, med = 99.5) for the intact prostate RapidPlan model, 99.9 ± 0.3 (min = 99.0, max = 101.3, med = 100.0) for prostate bed clinical plans, and 99.6 ± 0.3 (min = 98.7, max = 100.2, med = 99.6) for the prostate bed RapidPlan model. Overall, for plans generated via RapidPlan, an additional ∼0.5% in plan normalization relative to the clinical plans was necessary to achieve the main target coverage goal (D_95% _≥ 100%).

### 3.2 Plan evaluation metrics for CBCT‐based datasets

#### 3.2.1 RTOG‐based DVH metrics

**FIGURE 2:**
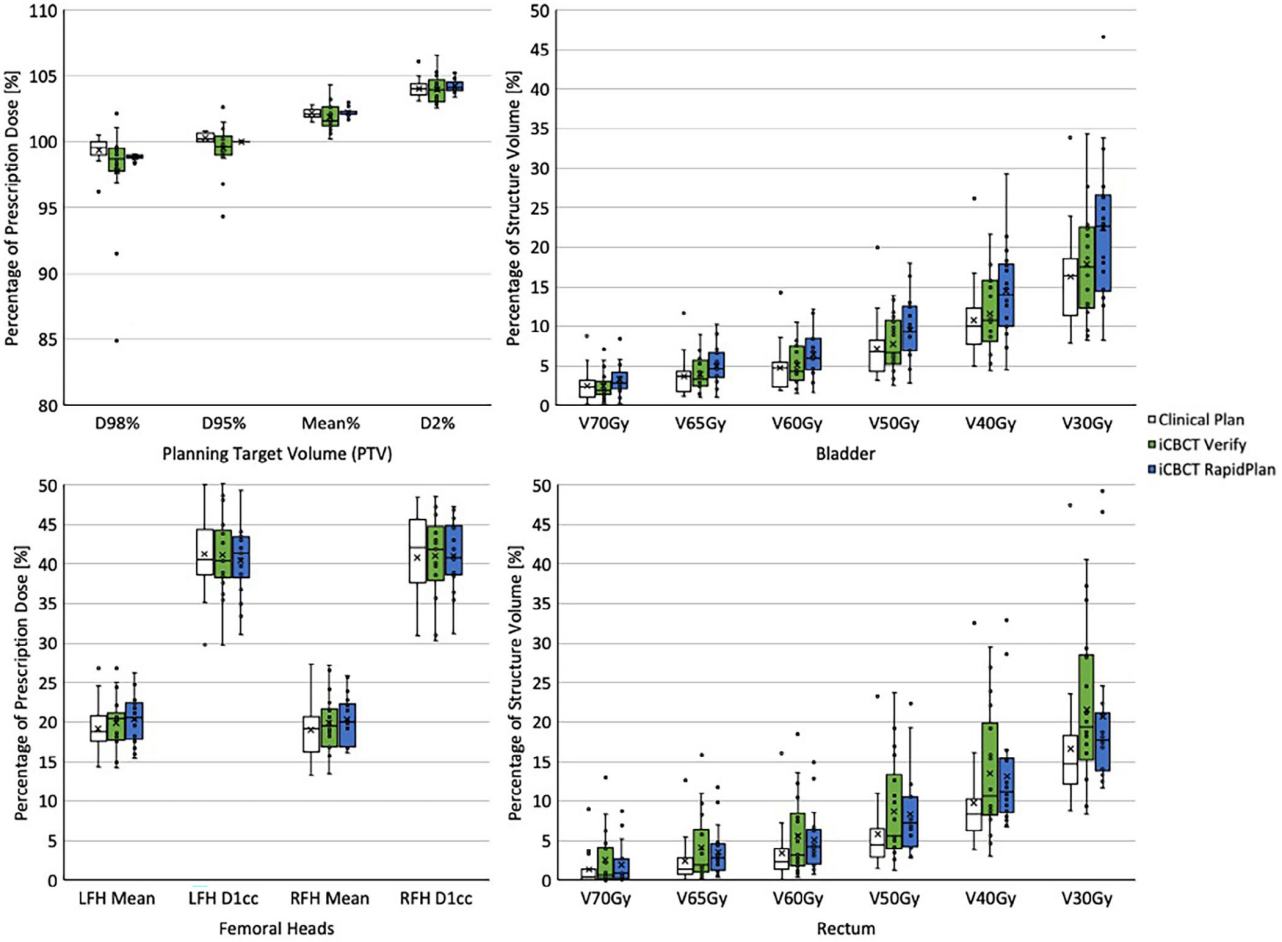
Distribution of relevant RTOG‐based dose‐volume histogram (DVH) metrics for the iCBCT‐based intact prostate cohort. Shown are DVH values for the intact prostate planning target volume (PTV), bladder, rectum, and left and right femoral heads (LFH and RFH). All volume‐based metrics are calculated as the equivalent dose in 2 Gy fractions to account for variable fractionation regimens within the group.

**FIGURE 3:**
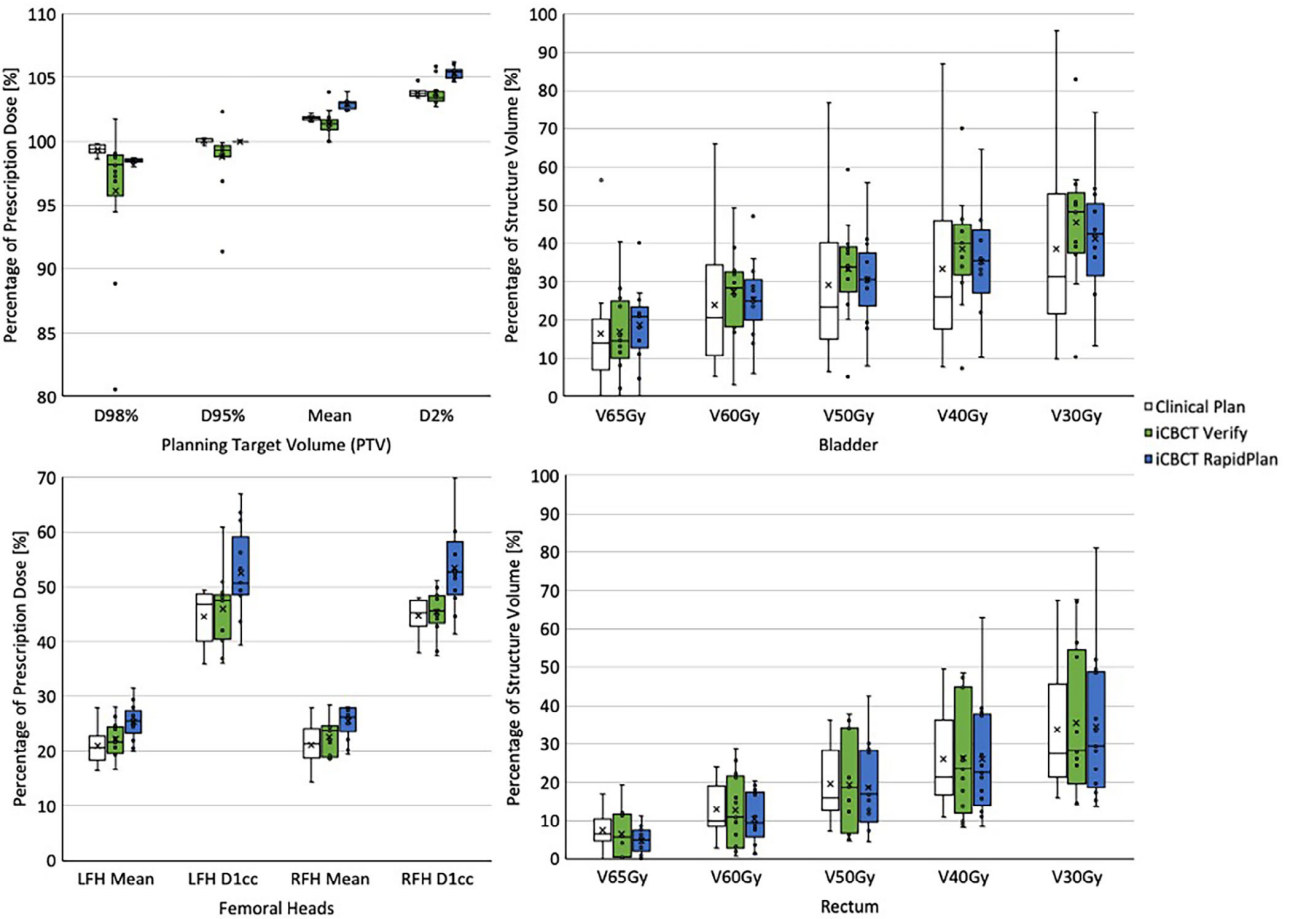
Distribution of relevant RTOG‐based dose‐volume histogram (DVH) metrics for the iCBCT‐based prostate bed cohort. Shown are DVH values for the prostate bed planning target volume (PTV), bladder, rectum, and left and right femoral heads (LFH and RFH). All volume‐based metrics are calculated as the equivalent dose in 2 Gy fractions to account for variable fractionation regimens within the group.

Mean values for the analyzed RTOG‐based DVH metrics for all three groups in the iCBCT‐based cohorts are presented in Table 5, while outcomes of the statistical analysis are provided in Tables A1 and A2 (in the Appendix) for intact prostate and prostate bed cases, respectively. Additionally, distributions of these metrics are depicted in Figure 2 (intact prostate) and Figure 3 (prostate bed). Of note, relative to the structure volumes from the intact prostate clinical plans on pCT, average volume differences of −2.2% ± 3.9% (intact prostate CTV), −30.3% ± 45.2% (bladder), and −8.8% ± 26.7% (rectum) were observed for the modified structure sets on iCBCT images due to tumor shrinkage and variation in organ filling. For prostate bed patients, these volume differences were −3.7% ± 2.7% (prostate bed CTV), −37.6% ± 37.3% (bladder), and −8.2% ± 33.3% (rectum).

**TABLE 5:** Mean RTOG‐based dose‐volume histogram (DVH) metric values for all cases within the iCBCT datasets. Values are reported to one standard deviation. Dose‐based metrics (e.g., D_98%_) are relative to the prescription dose, while volume‐based metrics (e.g., V_70Gy,EQD2_) are relative to the total volume of the corresponding organ.

	DVH parameter	Intact prostate (*n* = 20)			Prostate bed (*n* = 15)		
		Clinical	iCBCT verify	iCBCT RapidPlan	Clinical	iCBCT verify	iCBCT RapidPlan
PTV	D_98%_ [%]	99.4 ± 1.0	97.8 ± 3.8	98.9 ± 0.2	99.4 ± 0.4	96.1 ± 5.3	98.5 ± 0.2
	D_95%_ [%]	100.3 ± 0.3	99.5 ± 1.8	100.0 ± 0.0	100.1 ± 0.1	98.8 ± 2.4	100.0 ± 0.0
	D_mean_ [%]	102.1 ± 0.4	101.9 ± 1.0	102.2 ± 0.3	101.8 ± 0.2	101.4 ± 1.0	102.9 ± 0.4
	D_2%_ [%]	104.0 ± 0.7	104.0 ± 1.0	104.2 ± 0.2	103.8 ± 0.3	103.7 ± 0.9	105.3 ± 0.5
	HI	0.05 ± 0.02	0.06 ± 0.04	0.05 ± 0.01	0.04 ± 0.01	0.08 ± 0.05	0.07 ± 0.01
	CI	0.97 ± 0.01	0.91 ± 0.07	0.95 ± 0.01	0.96 ± 0.01	0.88 ± 0.08	0.95 ± 0.01
Bladder	V_70Gy, EQD2_ [%]	2.5 ± 2.1	2.4 ± 1.8	3.3 ± 1.9	–	–	–
	V_65Gy, EQD2_ [%]	3.7 ± 2.5	3.9 ± 2.1	5.0 ± 2.3	16.4 ± 13.4	17.1 ± 10.7	18.8 ± 9.7
	V_60Gy, EQD2_ [%]	4.7 ± 2.9	5.1 ± 2.5	6.4 ± 2.7	23.9 ± 16.3	27.3 ± 10.9	25.5 ± 9.8
	V_50Gy, EQD2_ [%]	7.2 ± 3.9	7.8 ± 3.3	9.6 ± 3.8	29.0 ± 18.2	33.2 ± 12.3	30.7 ± 11.3
	V_40Gy, EQD2_ [%]	10.7 ± 4.8	11.6 ± 4.6	14.4 ± 5.7	33.3 ± 20.3	38.5 ± 14.0	35.4 ± 12.8
	V_30Gy, EQD2_ [%]	16.3 ± 6.0	17.8 ± 6.8	22.4 ± 9.0	38.6 ± 22.2	45.4 ± 16.1	41.3 ± 14.4
	D_mean_ [%]	20.4 ± 5.9	22.4 ± 6.3	25.7 ± 7.5	42.6 ± 20.2	49.6 ± 14.5	46.3 ± 13.2
	D_0.03cc_ [%]	103.8 ± 1.0	103.7 ± 1.2	104.3 ± 0.9	104.3 ± 1.0	104.3 ± 1.4	105.8 ± 1.0
Rectum	V_70Gy, EQD2_ [%]	1.3 ± 2.3	2.6 ± 3.5	1.9 ± 2.5	–	–	–
	V_65Gy, EQD2_ [%]	2.4 ± 3.0	4.2 ± 4.3	3.5 ± 3.0	7.5 ± 4.6	6.5 ± 6.0	4.8 ± 3.2
	V_60Gy, EQD2_ [%]	3.4 ± 3.6	5.6 ± 5.0	5.0 ± 3.7	12.9 ± 6.7	12.6 ± 9.2	10.3 ± 6.2
	V_50Gy, EQD2_ [%]	5.8 ± 4.9	8.7 ± 6.3	8.3 ± 5.1	19.6 ± 9.5	19.4 ± 11.9	18.5 ± 10.5
	V_40Gy, EQD2_ [%]	9.8 ± 6.2	13.5 ± 7.8	13.1 ± 6.9	25.6 ± 12.5	26.2 ± 14.6	26.0 ± 14.6
	V_30Gy, EQD2_ [%]	16.6 ± 8.3	21.5 ± 9.3	20.7 ± 10.2	33.8 ± 16.7	35.4 ± 18.4	34.4 ± 18.4
	D_mean_ [%]	22.8 ± 6.0	27.2 ± 6.4	26.2 ± 6.0	38.7 ± 12.8	41.3 ± 13.5	39.2 ± 13.7
	D_0.03cc_ [%]	100.2 ± 5.9	102.2 ± 3.2	101.7 ± 3.0	103.8 ± 0.7	103.8 ± 1.4	104.0 ± 1.0
LFH	D_mean_ [%]	19.2 ± 3.2	19.8 ± 3.3	20.4 ± 2.9	21.1 ± 3.1	22.2 ± 3.0	24.5 ± 3.2
	D_1cc_ [%]	41.2 ± 4.9	41.1 ± 5.0	40.5 ± 4.4	44.5 ± 4.7	45.9 ± 6.4	52.5 ± 7.6
RFH	D_mean_ [%]	19.0 ± 3.3	19.8 ± 3.5	20.3 ± 2.9	21.2 ± 3.4	22.6 ± 3.0	25.4 ± 2.9
	D_1cc_ [%]	40.8 ± 5.3	41.0 ± 5.1	41.0 ± 4.2	44.8 ± 3.2	45.5 ± 4.0	53.5 ± 7.1

Abbreviations: CI, conformity index; HI, heterogeneity index; LFH, left femoral head; PTV, planning target volume; RFH, right femoral head.

Within the iCBCT‐based intact prostate cohort, nearly all PTV metrics (excluding CI), exhibited no statistically significant differences. However, the mean D_95%_ value for iCBCT Verify plans failed to attain the primary target coverage goal (≥100%), while the conformity of iCBCT RapidPlans was significantly improved relative to iCBCT Verify plans. Statistically significant disparities were demonstrated for most bladder metrics (excluding the maximum dose [D_0.03cc_]), as the iCBCT RapidPlans resulted in increased volume‐based dose metrics for this structure of interest (though all metrics were within desired constraints). Similar statistical results were observed for the rectum, though iCBCT RapidPlans slightly decreased average dosimetric values relative to iCBCT Verify plans (not statistically significant) and showed no statistical differences from clinical plans for a majority of the rectum metrics (6 of 8). No statistical variation was present within femoral head metrics.

Within the iCBCT‐based prostate bed cohort, nearly all PTV metrics (excluding D_95%_) exhibited statistically significant disparities among the three plans, as iCBCT RapidPlans demonstrated elevated values for D_mean_ and D_2%_. As before, iCBCT Verify plans narrowly failed to meet the desired target coverage goal (on average), while PTV coverage for iCBCT RapidPlans was significantly more conformal than for iCBCT Verify plans. Within bladder and rectum metrics, significant differences were observed only for the bladder D_0.03cc_, in which iCBCT RapidPlans exhibited a marginal hot spot increase on average. Femoral head doses were (statistically) significantly increased for iCBCT RapidPlans.

#### 3.2.2 PQM values

**FIGURE 4:**
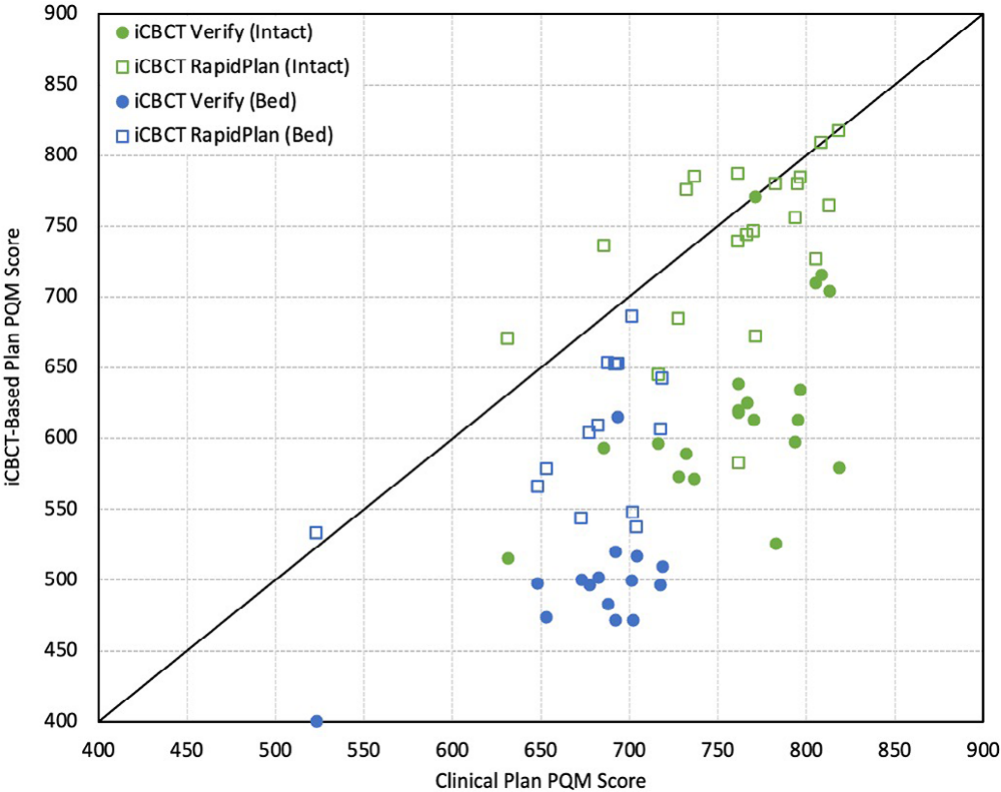
Plan quality metric (PQM) scoring comparison between clinical plans and iCBCT‐based plans for each cohort. Data points below the line indicate a higher PQM score for the clinical plan, while data points above the line indicate a higher PQM score for the iCBCT‐based plan (iCBCT Verify or iCBCT RapidPlan). Maximum achievable scores were 820 for intact prostate and 740 for prostate bed.

**FIGURE 5:**
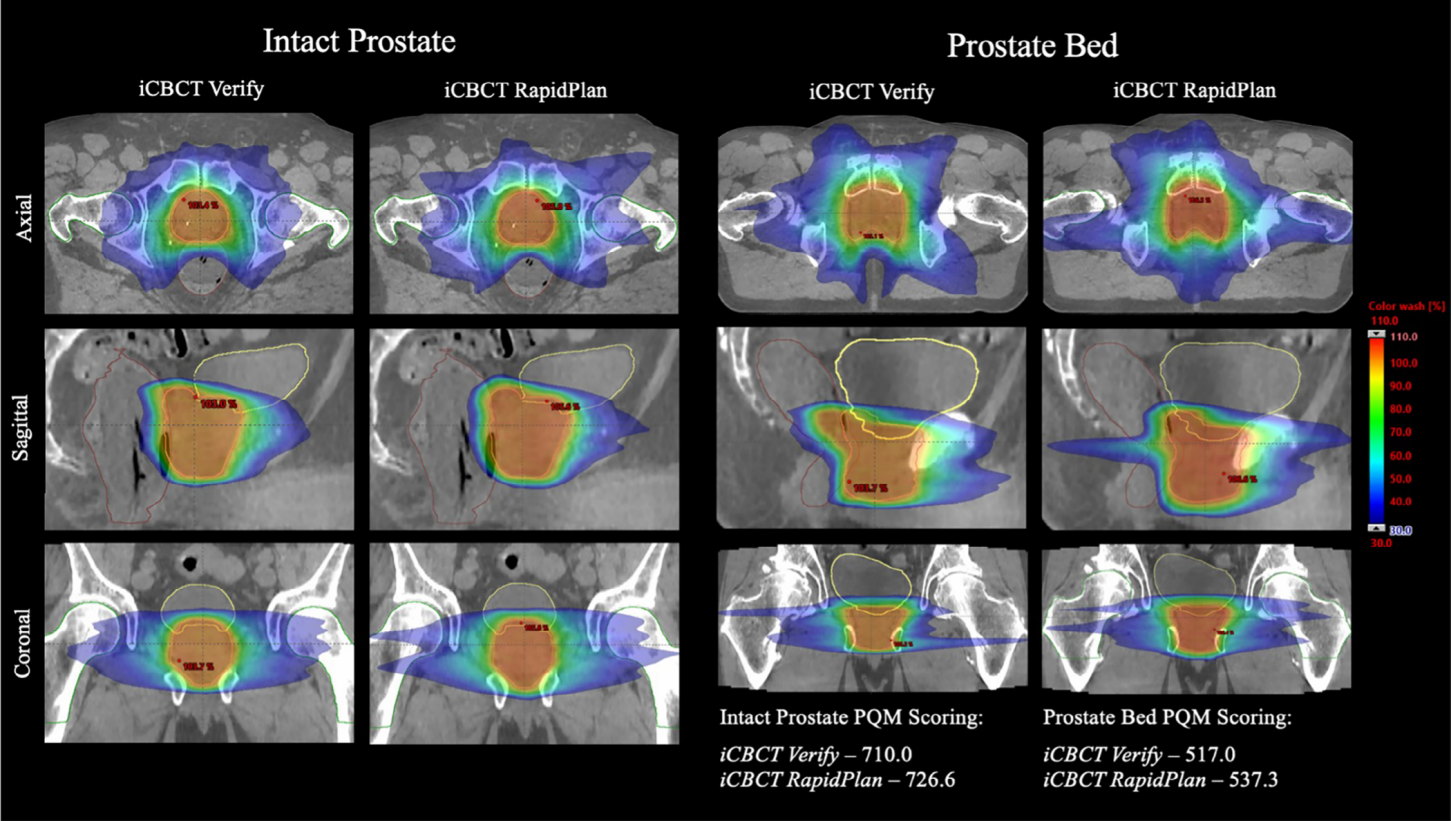
Comparison dose distributions with similar plan quality metric (PQM) scoring for each cohort of iCBCT Verify and iCBCT RapidPlan plans. Refined contours for relevant structure volumes are provided, including the planning target volume (pink), bladder (yellow), rectum (brown), and femoral heads (green). Color‐wash dose indicates the region of <110% and >30% of the prescribed dose.

Mean PQM values for the various plans are summarized in Tables 6 and 7, while these results can be visualized in Figure 4. Additionally, sample dose distributions for plans that achieved similar PQM scoring are demonstrated in Figure 5. Within the intact prostate cohort, iCBCT RapidPlans (mean total score of 739.0 ± 61.8) scored higher than iCBCT Verify plans (620.0 ± 65.2) and the original clinical plans (761.6 ± 48.0) in 90% of cases (18/20) and 30% of cases (6/20), respectively. Statistical tests (Freidman and Nemenyi) revealed no significant variation between clinical plans and iCBCT RapidPlans only (Nemenyi *p*‐value = 0.38). Overall, only 2 failures were observed for clinical plans (both for the target D_min_ sub‐metric), compared to 28 failures for iCBCT Verify plans (27 associated with target sub‐metrics), and 4 failures for iCBCT RapidPlans (3 linked to the target D_min_ sub‐metric).

**TABLE 6:** Mean plan quality metric (PQM) values for intact prostate cases (*n* = 20) within the iCBCT dataset. Values for the sub‐metrics and the corresponding sub‐metric scores are reported to one standard deviation. Failures indicate instances in which no points were awarded.

		Clinical plan			iCBCT verify plan			iCBCT RapidPlan		
	Sub‐metric	Mean value	Mean score	Fail	Mean value	Mean score	Fail	Mean value	Mean score	Fail
PTV	D_95%_ [%]	100.3 ± 0.3	100.0 ± 0.0	0	99.5 ± 1.8	22.0 ± 42.0	15	100.0 ± 0.0	100.0 ± 0.0	0
	D_0.03cc_ [%]	105.1 ± 0.8	92.8 ± 11.6	0	105.1 ± 1.1	89.4 ± 14.2	0	105.4 ± 0.8	89.7 ± 13.1	0
	Min [%]	92.2 ± 2.6	89.0 ± 31.0	2	85.8 ± 9.8	39.0 ± 49.0	12	91.4 ± 1.7	83.0 ± 37.0	3
Bladder	V_70Gy, EQD2_ [%]	0.6 ± 1.1	75.1 ± 9.1	0	0.6 ± 1.1	75.1 ± 8.9	0	0.8 ± 1.4	74.0 ± 11.1	0
	V_65Gy, EQD2_ [%]	3.7 ± 2.5	46.3 ± 2.5	0	3.9 ± 2.1	46.1 ± 2.1	0	5.0 ± 2.3	45.0 ± 2.3	0
	D_0.03cc_ [%]	103.8 ± 1.0	55.1 ± 19.9	0	103.7 ± 1.2	56.0 ± 23.7	0	104.3 ± 0.9	44.6 ± 20.0	1
Rectum	V_70Gy, EQD2_ [%]	1.3 ± 2.3	80.0 ± 0.0	0	2.6 ± 3.5	79.1 ± 3.5	0	1.9 ± 2.5	80.0 ± 0.0	0
	V_65Gy, EQD2_ [%]	2.4 ± 3.0	76.8 ± 3.9	0	4.2 ± 4.3	74.5 ± 5.7	0	3.5 ± 3.0	75.3 ± 4.1	0
	D_0.03cc_ [%]	100.2 ± 5.6	46.6 ± 5.5	0	102.2 ± 3.2	38.7 ± 15.6	1	101.7 ± 3.0	47.1 ± 6.4	0
LFH	V_50Gy, EQD2_ [cc]	0.0 ± 0.0	50.0 ± 0.0	0	0.0 ± 0.0	50.0 ± 0.0	0	0.0 ± 0.0	50.0 ± 0.0	0
RFH	V_50Gy, EQD2_ [cc]	0.0 ± 0.0	50.0 ± 0.0	0	0.0 ± 0.0	50.0 ± 0.0	0	0.0 ± 0.0	50.0 ± 0.0	0
	**Total score**:		**761.6 ± 48.0**			**620.0 ± 65.2**			**739.0 ± 61.8**	

Abbreviations: LFH, left femoral head; PTV, planning target volume; RFH, right femoral head.

**TABLE 7:** Mean plan quality metric (PQM) values for prostate bed cases (*n* = 15) within the iCBCT dataset. Values for the sub‐metrics and the corresponding sub‐metric scores are reported to one standard deviation. Failures indicate instances in which no points were awarded.

		Clinical plan			iCBCT verify plan			iCBCT RapidPlan		
	Sub‐metric	Mean value	Mean score	Fail	Mean value	Mean score	Fail	Mean value	Mean score	Fail
PTV	D_95%_ [%]	100.1 ± 0.1	93.0 ± 27.0	1	98.8 ± 2.4	6.7 ± 25.0	14	100.0 ± 0.0	100.0 ± 0.0	0
	D_0.03cc_ [%]	105.3 ± 0.7	92.9 ± 11.9	0	105.2 ± 1.1	89.8 ± 17.9	0	106.7 ± 0.8	65.8 ± 15.1	0
	Min [%]	93.0 ± 1.4	100.0 ± 0.0	0	79.9 ± 12.6	15.0 ± 36.0	12	90.7 ± 1.4	69.0 ± 46.0	4
Bladder	V_65Gy, EQD2_ [%]	16.4 ± 13.4	36.3 ± 11.2	0	17.1 ± 10.7	35.8 ± 8.9	0	18.8 ± 9.7	34.3 ± 8.1	0
	D_0.03cc_ [%]	104.3 ± 1.0	62.2 ± 12.9	0	104.3 ± 1.4	62.4 ± 17.8	0	105.8 ± 1.0	42.2 ± 12.8	1
Rectum	V_70Gy, EQD2_ [%]	0.0 ± 0.0	80.0 ± 0.0	0	0.0 ± 0.0	80.0 ± 0.0	0	0.0 ± 0.0	80.0 ± 0.0	0
	V_65Gy, EQD2_ [%]	7.5 ± 4.6	70.0 ± 6.1	0	6.5 ± 6.0	71.3 ± 8.0	0	4.8 ± 3.2	73.6 ± 4.3	0
	D_0.03cc_ [%]	103.8 ± 0.7	43.7 ± 5.5	0	103.7 ± 1.4	42.1 ± 9.0	0	104.1 ± 1.0	39.8 ± 4.4	0
LFH	V_50Gy, EQD2_ [cc]	0.0 ± 0.0	50.0 ± 0.0	0	0.0 ± 0.0	50.0 ± 0.0	0	0.1 ± 0.1	49.7 ± 1.2	0
RFH	V_50Gy, EQD2_ [cc]	0.0 ± 0.0	50.0 ± 0.0	0	0.1 ± 0.1	49.9 ± 0.4	0	0.1 ± 0.1	49.2 ± 1.2	0
	**Total score**:		**677.5 ± 49.0**			**496.6 ± 45.1**			**604.0 ± 54.8**	

Abbreviations: LFH, left femoral head; PTV, planning target volume; RFH, right femoral head.

In the prostate bed cohort, iCBCT RapidPlans (mean total score of 604.0 ± 54.8) demonstrated superior scoring relative to iCBCT Verify plans (496.6 ± 45.1) for all 15 cases. Compared to clinical plans (677.5 ± 49.0), a higher score for iCBCT RapidPlans was achieved in a single case, though variation in mean PQM scoring was not statistically significant between these plan types as before (Nemenyi *p*‐value = 0.08). Only one sub‐metric failure was observed for the original clinical plans (PTV D_95%_), whereas iCBCT Verify plans incurred 26 failures (all associated with target sub‐metrics) compared to 5 failures for iCBCT RapidPlans (4 related to the target D_min_ sub‐metric).

#### 3.2.3 Additional plan quality metrics

On average, iCBCT RapidPlans demonstrated a reduction in total MUs relative to the clinical and iCBCT Verify plans for both intact prostate (lower by −8.6% ± 9.3%; min = −23.2%, max = 7.6%, med = −7.7%) and prostate bed cases (lower by −15.6% ± 6.0%; min = −27.6%, max = −5.6%, med = 3.6%).

Average plan normalization values were 99.5 ± 0.5 (min = 97.8, max = 100.0, med = 99.7) for intact prostate clinical (and iCBCT Verify) plans, 97.6 ± 0.7 (min = 95.9, max = 98.7, med = 97.5) for intact prostate iCBCT RapidPlans, 100.0 ± 0.2 (min = 99.6, max = 100.4, med = 100.0) for prostate bed clinical (and iCBCT Verify) plans, and 98.1 ± 0.6 (min = 97.1, max = 99.3, med = 98.0) for prostate bed iCBCT RapidPlans. Relative to the original clinical plans, plans generated directly on iCBCT images via RapidPlan required an additional ∼2.0% in plan normalization to achieve the desired target coverage.

Within the in‐house oART software under development at this institution, the mean time required to generate and report metrics for iCBCT Verify plans [in mm:ss] was 02:38 ± 00:33 (min = 01:38, max = 03:25, med = 02:37) for intact prostate and 02:22 ± 00:10 (min = 02:10, max = 02:47, med = 02:21) for prostate bed. To generate and optimize the “adapted” iCBCT RapidPlans, the average time needed was 06:09 ± 02:06 (min = 03:47, max = 10:43, med = 05:11) for intact prostate and 07:12 ± 01:04 (min = 6:00, max = 09:35, med = 06:57) for prostate bed. When considering conventional (*n* = 12) versus hypo‐fractionated (*n* = 8) cases for the intact prostate cohort, a noticeable increase in the mean iCBCT RapidPlan generation time was observed for hypo‐fractionated cases (mean time of 08:41 ± 01:52 compared to 04:56 ± 00:58 for conventional cases), primarily attributable to variation in the utilized structure resolution for plan optimization based on institutional planning guidelines (1.5 mm for most hypo‐fractionated cases, 2.5 mm for conventional cases). In comparison, plan generation of intact prostate cases treated with two full treatment arcs (VMAT) takes approximately 13 min with the oART‐dedicated Varian Ethos optimization engine.^35^ Of note, when contrasted with VMAT optimization, the generation of static intensity‐modulated radiotherapy (IMRT) plans on the Ethos platform is markedly more rapid, with 12‐field IMRT prostate plans produced in approximately 5 min.^35^ This is marginally quicker than that reported for VMAT plan generation with the proposed approach in this study.

## 4 DISCUSSION

This investigation aimed to evaluate the practicality of utilizing institution‐specific RapidPlan models to generate clinically acceptable plans in the male pelvic region via single optimization on iCBCT datasets. To ensure satisfactory performance, models first underwent validation by comparing clinical and RapidPlan‐generated plans on pCT datasets. These results demonstrated that refined intact prostate and prostate bed models were capable of producing VMAT plans that were overall dosimetrically comparable to, or improved relative to (especially for target coverage), clinically‐treated plans iteratively optimized by a trained dosimetrist, with satisfactory performance exhibited across various treatment energies, prescriptions, and fractionation regimens. While similar validation studies have been conducted for intact prostate models,^24^, ^25^, ^26^, ^27^, ^28^, ^29^ this study is among the first to extensively analyze a prostate bed model in this manner.

Within the testing datasets, application of the intact prostate RapidPlan model slightly increased dose estimates for bladder and femoral head metrics while marginally improving rectum dose metrics relative to clinical plans. Bladder and rectum sparing were improved through utilization of RapidPlan for the prostate bed cohort (though not in a statistically significant manner), while femoral head dose was increased as before. Importantly, several sub‐metric failures in the PQM scoring process were detected in both the clinical and RapidPlan‐generated plans (see Tables 3 and 4), indicating that for a small proportion of the test cases, all ideal clinical constraints could not be met by either the manual planning or KBP approach. This does not inherently imply that such plans are suboptimal, however, as treatment planning is fundamentally a tradeoff between achieving adequate target coverage and appropriate normal tissue sparing. Thus, there will inevitably be scenarios in which all desired constraints cannot be satisfied contingent upon what is prioritized by the physician. Of note, the refined models as applied in this study represent the second iteration of trained RapidPlan models implemented at this institution for treatment involving the male pelvic region. Per clinical protocol, dosimetrists are asked to utilize the appropriate RapidPlan model when first generating intact prostate and prostate bed plans, followed by further refinement and optimization if necessary to adhere to clinical constraints. As a result, all plans used to train the models applied in this study were informed by previous RapidPlan models, limiting the influence of inter‐planner variability on model training and thus the quality of the reported results.

Following model validation, an adaptive scenario was retrospectively simulated for a select cohort of intact prostate and prostate bed patients, including comparison of various dose metrics between scheduled (iCBCT Verify) and adapted (iCBCT RapidPlan) plans. The primary objective was to assess the fidelity of automated treatment planning on iCBCT images to determine if adapted plans that potentially improve tumor coverage and/or normal tissue sparing could be generated via the proposed method. On‐treatment imaging data from the final fraction was selected in an effort to systematically introduce potentially large anatomical variations between pCT and iCBCT acquisitions to assess the functionality of this tool in instances where plan adaptation may likely be beneficial. However, conducting an in‐depth evaluation into the expected utility (and frequency of use) of this tool for oART through an exhaustive retrospective analysis of multiple fractions across a larger cohort of patients was beyond the scope of this study.

For nearly all cases, application of the appropriate RapidPlan model resulted in noticeably improved PQM scoring with markedly less sub‐metric failures when compared to the original plan as scheduled (Tables 6 and 7). Contrary to what was noted regarding PQM sub‐metric failures observed within the pCT‐based testing datasets, failures within these iCBCT Verify plans were primarily attributable to anatomical variations from the pCT on which these plans were originally generated and were not a result of conflicting constraints. Overall, iCBCT RapidPlan plans also demonstrated improved PTV coverage and conformality relative to iCBCT Verify plans. Utilizing RapidPlan was shown to slightly increase volumetric and point‐dose metrics for the bladder while generally improving rectal sparing for each patient cohort, though these differences were not statistically significant in most instances. Prostate bed plans generated via RapidPlan also exhibited increased femoral head dose (as evident in Figure 5), though clinical constraints were not exceeded in any case. On average, the required MUs for delivery of iCBCT RapidPlan plans were lower by 8.6% (intact prostate) and 15.6% (prostate bed) relative to the scheduled iCBCT Verify plans, thus reducing overall treatment delivery time (assuming equivalent dose rates) and lessening the probability of intra‐fractional patient motion during this portion of the process.

While direct use of RapidPlan on iCBCT datasets provided generally improved plan metric outcomes relative to iCBCT Verify, overall plan quality remained slightly inferior to that observed for original clinical plans on pCT datasets, and noticeably more plan normalization was necessary for iCBCT RapidPlan results to achieve the desired target coverage. However, this result was not unanticipated for several reasons. Most notably, the clinical plans (and thus those propagated for iCBCT Verify) were produced with utilization of various refined optimization structures through multiple iterations of the plan optimization process. For iCBCT RapidPlan‐generated plans, only target/OAR overlap volumes (for bladder and rectum) and a simple PTV optimization structure could be auto‐generated via the in‐house oART platform under development, and only a single optimization was performed due to the expeditious nature of the workflow as necessary. It is likely that the utilization of more complex/refined optimization structures for the original clinical plans played a significant role in the MU variation observed between the clinical and iCBCT‐based plans (MUs were reduced overall for the iCBCT‐based plans, suggesting slightly less modulation). Additionally, this institution no longer mandates a full bladder for treatments of this nature as an internal investigation revealed minimal impact on dose delivery accuracy and a noticeable degradation in patient comfort. Instead, protocols have been revised to ensure that some minimum bladder volume is achieved prior to treatment initiation, and thus there can be pronounced variation in daily bladder filling. For the iCBCT‐based cohorts in this study, average total bladder volumes observed on the final fraction iCBCT images were reduced by 30.3% (intact prostate) and 37.6% (prostate bed) relative to the original pCT. Provided this substantial discrepancy, for the same treated volume on pCT and iCBCT, volume‐based metrics for the bladder (e.g., V_70Gy,EQD2_) would be expected to be higher for iCBCT since the overall structure volumes were noticeably smaller (and these metrics are relative to the total volume of the corresponding organ). In most instances (excluding a single PQM sub‐metric failure in each cohort), all bladder metrics for iCBCT RapidPlan‐generated plans still satisfied desired clinical constraints. Lastly, variation in achievable image quality between pCT and iCBCT also contributes some to differences observed among the various plans, though this impact has been mitigated somewhat by enhanced software development. Previous studies on iCBCT have demonstrated that application of this refined reconstruction algorithm significantly improves image quality compared to the traditional filtered‐back projection approach.^36^, ^37^, ^38^ Furthermore, comparison of Hounsfield Unit (HU) values between iCBCT and pCT revealed a variation of less than 30 HU for nearly all material plugs evaluated, yielding a DVH metric accuracy in the male pelvic region within 0.5% (for target clinical coverage) and 1.0% (OARs) relative to that achieved with pCT (with utilization of the standard pCT‐based HU to electron density calibration) as demonstrated in both phantom and patient studies.^37^ As comprehensive internal investigations have also been conducted to validate iCBCT for dose computation, the calculation accuracy relative to pCT has proven to be more than sufficient for the proposed application. Moreover, the integration of the HyperSight (Varian Medical Systems, Inc.) advanced imaging solution on conventional TrueBeam linacs is expected to further improve plan generation and dose calculation accuracy. Currently available on Halcyon/Ethos platforms, recent studies have demonstrated that HyperSight is capable of acquiring CBCT images with an overall image quality comparable to that achieved with CT simulation systems and further improved relative to existing CBCT imagers.^39^, ^40^, ^41^ This clinic intends to incorporate this technology within the coming year, with minimal anticipated changes to the proposed workflow (note that HyperSight images on TrueBeam will be able to be acquired 2× faster than iCBCT). Nonetheless, despite the noted limitations in the proposed approach, mean total PQM values for clinical and iCBCT RapidPlan‐generated plans exhibited no statistically significant variation for either intact prostate or prostate bed cases.

Complimentary to this preliminary feasibility study, further investigation is imperative to fully comprehend the efficacy of this tool within the anticipated patient cases at this institution and to identify additional treatment sites for which the proposed method may exhibit clinical utility. Although this study focused on intact prostate and prostate bed, KBP with RapidPlan has demonstrated promising results across a variety of anatomical sites, including lung and head and neck (H&N).^42^, ^43^ Further studies will be conducted to determine the feasibility of applying this approach to these sites. Of note, the extension of the proposed solution to lung and H&N oART will necessitate the refinement of additional KBP and auto‐segmentation models, with the efficacy of such processes likely further improved as well through incorporation of the HyperSight solution. Moreover, considering the increased staff involvement required for such a workflow, it is possible that clinical implementation of this approach will be prioritized for hypo‐fractionated regimens (e.g., prostate SBRT) that limit the total number of treatment sessions. Finally, although not pertinent to cases included in this specific study, there may be scenarios in which it is advantageous to consider the bowel metrics in the iCBCT‐based PQM scoring as well, particularly if this methodology is extended to the treatment of patients with nodal or metastatic involvement, where there is an increased probability of overlap between the target volume and the large or small bowel. Therefore, additional DLAS model training for these structures will be necessary, or the overlapping regions will need to be manually delineated on a case‐by‐case basis (should such instances occur infrequently).

Importantly, standard protocols must be developed and refined to facilitate the decision to perform plan adaption or not based on quantitative and qualitative evaluation/comparison. For example, while numerous failures in PTV coverage (based on PQM sub‐metric values) were noted for iCBCT Verify plans, most failing metrics were still within 1%−2% of the desired value. Additionally, under normal image‐guided treatment, it is not expected that the PTV would be fully covered on any given fraction as this volume is simply a tool to ensure full coverage to the CTV. Thus, failure in these metrics does not necessarily indicate that a given patient will benefit from plan adaptation. Criteria to aid in the discernment of which plan (scheduled or adapted) should be utilized and/or if plan adaptation should even occur will be further delineated as the development of this in‐house oART tool progresses toward clinical integration within the coming months.

Furthermore, additional components of the proposed workflow that extend the duration required for procedural completion need to be considered, such as the execution of physics checks and patient‐specific quality assurance (PSQA). At this institution, secondary plan verification and PSQA for treatments of this type are accomplished through application of a commercial software solution (Mobius3D, Varian Medical Systems, Inc.). The physics second check process can be automated utilizing Mobius3D, while PSQA can be performed via log‐file analysis with the MobiusFX package. While PSQA is typically implemented prospectively, the proposed workflow incorporates PSQA of the adapted plan retrospectively following treatment administration (with the initial plan PSQA still conducted prior to treatment initiation). Per investigation, this aligns with what is typically performed by other facilities using CBCT‐based oART solutions (e.g., Ethos). Given the automation capabilities for both of these activities, it is anticipated that the quality assurance processes will not impose significant additional time requirements to this workflow.

One prominent limitation in performing oART with any platform is the potential instability of the patient's internal anatomy as the adaptive workflow progresses following image acquisition. For example, continued bladder filling and mobility of gas in the bowel and/or rectum can induce variability even within the relatively brief timeframe required to complete this procedure, rendering the full clinical benefit of this process not fully realized. For the proposed method, a second iCBCT image will be acquired immediately preceding treatment administration to revalidate patient setup and anatomy, and it is anticipated that further deformation may occur to some (hopefully limited) extent during the timeframe required to complete the oART process. The incorporation of automation when possible to curtail the necessary workflow duration in addition to refined fixation techniques (e.g., exploring utilization of a rectal balloon for all oART patients) and patient education materials (e.g., instructions and/or procedures on how to best achieve stable bladder filling fraction‐to‐fraction), for example, aim to mitigate this issue to the greatest extent possible. Such process considerations will be further elaborated upon in a future study, as addressing these concerns is imperative to ensuring the applicability of this solution. Nonetheless, the results of this investigation underscore the viability of leveraging RapidPlan to expeditiously generate clinically acceptable plans adapted to the daily anatomy as observed on iCBCT images. Moreover, the time required to generate plans of this nature (VMAT, two full treatment arcs) is reduced by nearly a factor of two when compared to the oART‐dedicated Varian Ethos platform, further accentuating the practicality of the proposed approach.

## 5 CONCLUSION

This study demonstrated the practicality of utilizing a commercial KBP tool to produce clinically acceptable intact prostate and prostate bed VMAT plans on iCBCT (and pCT) image sets within a single optimization. Model validation and testing on pCT datasets confirmed that plans created via RapidPlan were overall dosimetrically comparable to, or superior to, clinical plans iteratively refined by a trained dosimetrist. The models were then retrospectively utilized to generate adapted plans on iCBCT datasets for select intact prostate and prostate bed patients. Direct application of RapidPlan on iCBCT produced satisfactory intact prostate and prostate bed plans with generally improved PTV coverage/conformality and rectal sparing relative to the scheduled plan, though bladder dose metrics were marginally increased on average (but still within clinical constraints for nearly all cases). Furthermore, the time required to generate the adapted VMAT plans via the proposed method was reduced by nearly a factor of two relative to a popular CBCT‐based oART‐dedicated platform (Ethos). The ability to efficiently produce optimized clinical treatment plans on daily iCBCT images is an integral workflow component of the in‐house oART platform for conventional linacs under development at this institution. The results of this study suggest that for the male pelvic region, the proposed approach is a viable method by which to accomplish this essential and time‐sensitive task.

## AUTHOR CONTRIBUTIONS

**Riley C. Tegtmeier**: Conceptualization; data curation; formal data and statistical analysis; investigation; methodology; and writing—original draft. **Edward L. Clouser**: Conceptualization; scripting and automation; model training; data extraction; data curation; investigation; methodology; writing—review; and editing. **Brady S. Laughlin**: data preparation and review; investigation; writing—review; and editing. **Diego Santos Toesca**: data preparation and review; investigation; writing—review; and editing. **Mattison J. Flakus**: Data extraction; data curation; investigation; methodology; writing—review; and editing. **Sara Bashir**: Data extraction; data curation; investigation; methodology; writing—review; and editing. **Christopher J. Kutyreff**: data preparation and review; investigation; methodology; writing—review; and editing. **Dean Hobbis**: data preparation and review; investigation; methodology; writing—review; and editing. **Daniel P. Harrington**: model training; investigation; methodology; writing—review; and editing. **Jennifer L. Smetanick**: Data preparation; data curation; model training; writing—review; and editing. **Nathan Y. Yu**: Consultation; data review; writing—review; and editing. **Carlos E. Vargas**: Consultation; data review; writing—review; and editing. **Sarah E. James**: Consultation; data review; writing—review; and editing. **Jean—Claude M. Rwigema**: Consultation; data review; writing—review; and editing. **Yi Rong**: Conceptualization; supervision; investigation; methodology; writing—primary review; and editing.

## CONFLICT OF INTEREST STATEMENT

The authors declare no conflicts of interest.

## ETHICS STATEMENT

This study received approval by the Mayo Clinic Institutional Review Board (IRB ID: 22‐002186).

## Data Availability

Research data that support the findings of this article will be shared upon reasonable request to the corresponding author.

## 1

See Tables A1 and A2 for statistical analysis results comparing relevant RTOG‐based DVH and PQM metrics for the iCBCT‐based intact prostate and prostate bed cohorts, respectively.

**TABLE A1:** Statistical analysis results for the intact prostate cohort (*n* = 20) in the iCBCT dataset. Mean RTOG‐based dose‐volume histogram values were compared via ANOVA single‐factor and Tukey's honestly significant difference (HSD) post‐hoc tests, while mean plan quality metric (PQM) values were compared via Freidman and Nemenyi tests (values shown in italics). *P*‐values shown in bold indicate those that demonstrate no statistically significant variation.

			Tukey's HSD (*Nemenyi*) *p*‐value*		
	Parameter	ANOVA (*Friedman*) *p*‐value*	Clinical/iCBCT verify	Clinical/iCBCT RapidPlan	iCBCT verify/iCBCT RapidPlan
PTV	D_98%_ [%]	**0.11**	**0.10**	**0.73**	**0.36**
	D_95%_ [%]	**0.08**	**0.07**	**0.67**	**0.32**
	D_mean_ [%]	**0.38**	**0.65**	**0.87**	**0.35**
	D_2%_ [%]	**0.74**	**0.98**	**0.84**	**0.74**
	HI	**0.20**	**0.23**	**0.30**	**0.99**
	CI	<0.01	<0.01	**0.42**	0.04
Bladder	V_70Gy, EQD2_ [%]	<0.01	**0.97**	0.01	<0.01
	V_65Gy, EQD2_ [%]	<0.01	**0.76**	<0.01	0.02
	V_60Gy, EQD2_ [%]	<0.01	**0.69**	<0.01	0.03
	V_50Gy, EQD2_ [%]	<0.01	**0.68**	<0.01	0.03
	V_40Gy, EQD2_ [%]	<0.01	**0.68**	<0.01	0.03
	V_30Gy, EQD2_ [%]	<0.01	**0.67**	<0.01	0.03
	D_mean_ [%]	<0.01	**0.37**	<0.01	**0.08**
	D_0.03cc_ [%]	**0.11**	**0.91**	**0.24**	**0.11**
Rectum	V_70Gy, EQD2_ [%]	**0.05**	0.04	**0.50**	**0.36**
	V_65Gy, EQD2_ [%]	0.04	0.03	**0.21**	**0.60**
	V_60Gy, EQD2_ [%]	0.03	0.03	**0.11**	**0.78**
	V_50Gy, EQD2_ [%]	0.01	0.02	**0.05**	**0.92**
	V_40Gy, EQD2_ [%]	0.01	0.02	0.03	**0.95**
	V_30Gy, EQD2_ [%]	0.01	0.02	**0.06**	**0.86**
	D_mean_ [%]	<0.01	<0.01	0.01	**0.64**
	D_0.03cc_ [%]	**0.32**	**0.32**	**0.51**	**0.93**
LFH	D_mean_ [%]	**0.71**	**0.99**	**0.72**	**0.78**
	D_1cc_ [%]	**0.08**	**0.39**	**0.06**	**0.57**
RFH	D_mean_ [%]	**0.96**	**0.98**	**0.96**	**0.99**
	D_1cc_ [%]	**0.06**	**0.16**	**0.05**	**0.50**
*Intact prostate PQM*		*<0.01*	*<0.01*	***0.38***	*<0.01*

*Notes*: **P*‐values for all tests based on an alpha value of 0.05, null hypothesis that there is no statistically significant difference between the mean values—if *p*‐value is <0.05, reject the null hypothesis.

Abbreviations: CI, conformity index; HI, heterogeneity index; LFH, left femoral head; PTV, planning target volume; RFH, right femoral head.

**TABLE A2:** Statistical analysis results for the prostate bed cohort (*n* = 15) in the iCBCT dataset. Mean RTOG‐based dose‐volume histogram values were compared via ANOVA single‐factor and Tukey's honestly significant difference (HSD) post‐hoc tests, while mean plan quality metric (PQM) values were compared via Freidman and Nemenyi tests (values shown in italics). *P*‐values shown in bold indicate those that demonstrate no statistically significant variation.

			Tukey's HSD (*Nemenyi*) *p*‐value*		
	Parameter	ANOVA (*Friedman*) *p*‐value*	Clinical/iCBCT verify	Clinical/iCBCT RapidPlan	iCBCT verify/iCBCT RapidPlan
PTV	D_98%_ [%]	0.04	0.04	**0.76**	**0.15**
	D_95%_ [%]	**0.05**	**0.08**	**0.99**	**0.10**
	D_mean_ [%]	<0.01	**0.30**	<0.01	<0.01
	D_2%_ [%]	<0.01	**0.99**	<0.01	<0.01
	HI	<0.01	**0.99**	<0.01	<0.01
	CI	<0.01	<0.01	**0.97**	<0.01
Bladder	V_65Gy, EQD2_ [%]	**0.44**	**0.93**	**0.43**	**0.64**
	V_60Gy, EQD2_ [%]	**0.28**	**0.25**	**0.72**	**0.67**
	V_50Gy, EQD2_ [%]	**0.16**	**0.14**	**0.72**	**0.47**
	V_40Gy, EQD2_ [%]	**0.09**	**0.07**	**0.62**	**0.38**
	V_30Gy, EQD2_ [%]	0.03	0.02	**0.48**	**0.22**
	D_mean_ [%]	0.01	0.01	**0.22**	**0.31**
	D_0.03cc_ [%]	<0.01	**0.98**	<0.01	<0.01
Rectum	V_65Gy, EQD2_ [%]	**0.33**	**0.39**	**0.99**	**0.39**
	V_60Gy, EQD2_ [%]	**0.06**	**0.63**	**0.05**	**0.28**
	V_50Gy, EQD2_ [%]	**0.17**	**0.97**	**0.19**	**0.28**
	V_40Gy, EQD2_ [%]	**0.82**	**0.99**	**0.82**	**0.88**
	V_30Gy, EQD2_ [%]	**0.99**	**0.99**	**0.99**	**0.99**
	D_mean_ [%]	**0.77**	**0.75**	**0.96**	**0.89**
	D_0.03cc_ [%]	**0.28**	**0.29**	**0.96**	**0.42**
LFH	D_mean_ [%]	<0.01	**0.51**	<0.01	<0.01
	D_1cc_ [%]	<0.01	**0.11**	<0.01	<0.01
RFH	D_mean_ [%]	<0.01	**0.86**	<0.01	<0.01
	D_1cc_ [%]	<0.01	**0.13**	<0.01	<0.01
*Prostate bed PQM*		*<0.01*	*<0.01*	***0.08***	*0.02*

*Notes*: **P*‐values for all tests based on an alpha value of 0.05, null hypothesis that there is no statistically significant difference between the mean values—if *p*‐value is <0.05, reject the null hypothesis.

Abbreviations: CI, conformity index; HI, heterogeneity index; LFH, left femoral head; PTV, planning target volume; RFH, right femoral head.
